# Spontaneous diseases in captive ratites (*Struthioniformes*) in northwestern Germany: A retrospective study

**DOI:** 10.1371/journal.pone.0173873

**Published:** 2017-04-12

**Authors:** Aimara Bello, Samuel Frei, Martin Peters, Anne Balkema-Buschmann, Wolfgang Baumgärtner, Peter Wohlsein

**Affiliations:** 1 Department of Pathology, University of Veterinary Medicine Hannover Foundation, Hannover, Lower Saxony, Germany; 2 Zoo Wuppertal, Wuppertal, North Rhine-Westphalia, Germany; 3 Chemisches und Veterinäruntersuchungsamt Westfalen, Arnsberg, North Rhine-Westphalia, Germany; 4 Institute of Novel and Emerging Infectious Diseases, Friedrich-Loeffler-Institute, Greifswald, Insel Riems, Mecklenburg-Western Pomerania, Germany; US Geological Survey, UNITED STATES

## Abstract

A retrospective study was carried out to define the spectrum of spontaneous diseases in ostriches and few other captive ratites, order *Struthioniformes*, in northwestern Germany. The investigation included 71 ratites necropsied between 1968 and 2014. They consisted of 54 ostriches, 5 emus, and 12 rheas with 37 adults, 23 juveniles and 11 neonates and embryonated eggs. Necropsy reports were reviewed, histologic preparations were re-examined and additional histochemical and immunohistochemical stains were carried out in selected cases. In many animals more than one morphologic diagnosis attributable to different disease processes was found. In adult animals (n = 37), the most commonly altered organ systems were the musculoskeletal system (49%), the digestive system (46%), and the cardiovascular system (46%) affected by traumatic lesions, inflammatory and degenerative changes, respectively. A spongy degeneration was found in the brain (35%); however, immunohistochemistry and western blotting failed to detect pathological prion protein. In juvenile animals (n = 23), the musculoskeletal (44%) and the digestive system (43%) were mainly affected by traumatic and inflammatory lesions, respectively. In embryonated eggs and neonates (n = 11) the major cause of death was circulatory failure associated with generalized subcutaneous edema as described for improper incubation conditions (64%). Summarized, most of the findings observed in adult and juvenile ratites in northwestern Germany are related to trauma, inflammatory and degenerative disorders, whereas death in embryonated eggs and neonates was most likely related to breeding conditions. A spongy encephalopathy awaits further studies to elucidate cause and pathogenesis.

## Introduction

Common ratite species kept in Germany are the ostrich (*Struthio camelus*), emu (*Dromaius novaehollandiae*) and Greater rhea (*Rhea Americana*). Ratites are very popular animals in zoological collections and are often housed in mixed exhibit enclosures with other species. Although the conservation status of most ratites is listed as least concerned [1], several European zoos are participating in breeding programs. Ostrich farming became very popular in Germany at the end of the 1980s with very high prices for breeding animals. Between 1994 and 1995 prices collapsed partly related to stricter husbandry and welfare regulations and partly caused by the upcoming evidence that ostrich farming is not as lucrative as previously assumed [2]. Since then, ostrich meat never played a considerable role in the German meat industry but has gained momentum at the beginning of this century as an alternative to beef in light of the BSE crisis and antibiotic use in cattle [3]. Apart from meat production, ostriches are also commercially raised as a source of leather, eggs and feathers [4]. In Germany, the total number of commercial and hobby breeding farms for ostriches is assumed to be about 120 to 150 [5], while other ratite species are not raised at any level of intensity and are mainly seen in zoological institutions and on private hobby farms. Emu farming has become very popular in India and USA [6] and emu products have attracted attention as alternate medical drugs [7, 8].

Frequent infectious and non-infectious spontaneous diseases in ostriches and other ratites have been reviewed [9–19], describing diseases that occur mostly in ratites farmed in countries in and outside of their areas of origin. It was noted that commercialization of ratite production goes along with increased disease frequency and parasitism [14]. This increment was related at least partially to improper management of farmed ratites in most cases [20]. Reports about spontaneous diseases of ratites in Germany are scarce. They include predominantly disorders related to husbandry including fractures, hematomas, joint luxations, ingested foreign bodies, perosis, persistent yolk sac, gastro-intestinal bacterial infections and respiratory mycoses [21]. In ostriches, gastritis caused by *Libyostrongylus douglassii* [22] and a spongiform-like encephalopathy have been reported [23, 24]. In rheas, a typhlocolitis associated with *Brachyspira hyodysenteriae* has been described [25].

Due to the increase and development of ostrich farming in Germany since the late 1980s and the lack of literature about ratite diseases kept in German zoos, this retrospective analysis was performed to get an overview about diseases and their frequency predominantly in kept ostriches in northwestern Germany. Morphological findings will be analyzed with respect to the age groups and will be discussed in the light of the etiology and possible impacts of husbandry. Furthermore, single rare and salient lesions will be highlighted which might be valuable in the light of comparative pathology.

## Material and methods

### Case records

The study was conducted on 71 ratites (case Nos. 1–71) of the order *Struthioniformes* including 54 ostriches (*Struthio camelus*), 5 emus (*Dromaius novaehollandiae*) and 12 rheas (*Rhea americana*) kept in northwestern Germany that were submitted for necropsy between 1968 and 2014. All animals were necropsied either at the Department of Pathology, University of Veterinary Medicine Hannover, Germany, or at the Chemisches und Veterinäruntersuchungsamt Westfalen, Arnsberg, North Rhine-Westfalia, Germany, where paraffin blocks of tissues are stored that are not publicly accessible. The records on medical history, animal data (gender, age at time of death), macroscopic and histological findings were analyzed. Furthermore, available results of additional investigations, such as immunohistological, parasitological, microbiological and virological investigations were included. The body condition was scored as good, moderate, poor and cachectic. Animals in a good body condition revealed abundant fat tissue within the furcular and abdominal regions whereas animals with a moderate body condition showed reduced corporal fat tissue. Animals in a poor body condition possessed only small fat reserves frequently associated with mild pectoral muscle atrophy. Cachectic animals lacked fat reserves and displayed serous atrophy of the coronary fat associated with moderate to severe atrophy of the pectoral muscle. Detailed information about the investigated animals including case number, animal identification number, species, age group, sex, nutritional status, husbandry, manner of death and major pathologic findings is given in Table 1. Postmortem examinations were carried out to investigate the cause of illness or death by order of the animals’ owner. All animals in this study that did not die spontaneously, were euthanized for humane reasons due to the severity of the illness or injury. Euthanasia was performed by the submitting veterinarian. This study was carried out in accordance to the German animal welfare act. All animals were dead at the time of submission and the authors confirm that no animal was sacrificed for the purpose of this study.

**Table 1:** Case number, animal ID-number, species, age group, sex, nutritional status, husbandry, manner of death and diagnosis of 71 ratites necropsied between 1968 and 2014.

Case number	Animal ID-number	Species	Age group	Sex	Nutritional status	Husbandry	Manner of death	Diagnosis
1	187–68	Ostrich	A	F	n. r.	Zoo	n. r.	Pneumonia (Aspergillosis) enteritis;
2	1172–68	Ostrich	A	F	Good	Zoo	n. r.	Visceral gout; tubulonephrosis; degenerative cardiomyopathy;
3	75–70	Ostrich	A	M	Good	Zoo	n. r.	Subcutaneous and muscular hemorrhage (neck); hepatic lipidosis; testicular atrophy;
4	1425–70	Ostrich	J	M	n. r.	Zoo	Spontaneous	Spine fracture;
5	4194–76	Ostrich	A	M	Good	Zoo	Spontaneous	subcutaneous and muscular hemorrhage (neck);
6	5023–77	Ostrich	A	F	Good	Zoo	Euthanasia	Enteritis; hydropericardium; degenerative vasculopathy; myositis;
7	6347–86	Ostrich	A	F	Good	Zoo	n. r.	Spongy degeneration (brain); degenerative vasculopathy;
8	3755–88	Ostrich	A	F	Moderate	Zoo	n. r.	Spongy degeneration (brain); tarsitis; arthrosis; degenerative vasculopathy;
9	527–89	Ostrich	J	M	Good	Zoo	Spontaneous	Encephalomalacia; sepsis with DIC (Salmonella); gastritis;
10	5345–89	Ostrich	J	F	Cachectic	Zoo	Spontaneous	Cachexia;
11	5202–89	Ostrich	J	M	Good	Zoo	n. r.	Suppurative pododermatitis; hepatitis;
12	5971–89	Ostrich	J	M	Moderate	Zoo	Euthanasia	Perosis; spongy degeneration (brain);
13	27–90	Ostrich	A	F	Good	Zoo	n. r.	Laceration of abdominal wall; cardiomyopathy;
14	313–90	Ostrich	J	n. r.	Moderate	Zoo	Euthanasia	Perosis;
15	2640–92	Ostrich	J	M	Poor	Zoo	Spontaneous	Rickets; multiple rib fractures; subcutaneous phlegmon;
16	223–93	Rhea	J	F	Good	Zoo	Spontaneous	Spine fracture;
17	171–93	Ostrich	A	F	Moderate	Zoo	Spontaneous	Spongy degeneration (brain); aerosacculitis; hepatosis;
18	440–93	Emu	A	F	Good	Zoo	Spontaneous	Fibrino-purulent serositis (intestine); spongy degeneration (brain);
19	441–93	Ostrich	A	M	Good	Zoo	Euthanasia	Spongy degeneration (brain); degenerative vasculopathy;
20	2504–94	Ostrich	A	M	Good	Zoo	n. r.	Spongy degeneration (brain); degenerative vasculopathy;
21	3240–97	Emu	A	F	Poor	Farm	Spontaneous	Rupture of flexor tendon with subcutaneous hemorrhage (tibiotarsus); acute meningeal and cerebellar hemorrhage; hepatitis; nephritis;
22	1830–97	Rhea	A	F	Good	Zoo	Spontaneous	Necrosis liver and spleen (Clostridiosis); degenerative vasculopathy;
23	101–02	Ostrich	A	M	Good	Zoo	Spontaneous	Rupture of limb musculature; degenerative vasculopathy;
24	1243–05	Rhea	N	n. r.	n. r.	Zoo	Spontaneous	Perforation of the gizzard; serositis;
25	1244–05	Rhea	N	n. r.	n. r.	Zoo	Spontaneous	no significant morphologic lesions
26	1531–05	Rhea	J	M	Poor	Zoo	Euthanasia	Osteosclerosis;
27	2341–05	Rhea	J	M	Good	Zoo	Euthanasia	Plexus chorioiditis; endoparasitism;
28	104–07	Rhea	A	M	Moderate	Zoo	Euthanasia	Skeletal muscle degeneration; subcutaneous hemorrhage (femur and neck);
29	646–07	Ostrich	J	M	Cachectic	Zoo	Spontaneous	Cachexia; Spongy degeneration (brain); tubulonephrosis;
30	647–07	Ostrich	J	M	Cachectic	Zoo	Spontaneous	Cachexia; Mycotic rhinitis, esophagitis and serositis; spongy degeneration (brain);
31	1247–07	Rhea	J	n. r.	Good	Farm	Spontaneous	Typhlocolitis (*Brachyspira* sp.);
32	121–09	Ostrich	A	F	Good	Zoo	Spontaneous	Fracture with subcutaneous hemorrhage (tibio-tarsus); hepatic lipidosis;
33	399–09	Ostrich	A	F	Good	Zoo	Spontaneous	No significant morphologic lesions contributing to death; degenerative vasculopathy;
34	1399–09	Ostrich	A	F	Poor	Zoo	Euthanasia	Disseminated granulomatous inflammation (Mycobacteriosis, Aspergillosis); spongy degeneration (brain); conjunctivitis;
35	376–10	Ostrich	E	n. r.	n. r.	Zoo	Spontaneous	Anasarca, omphalitis;
36	377–10	Ostrich	E	n. r.	n. r.	Zoo	Spontaneous	Anasarca, omphalitis;
37	396–10	Ostrich	N	M	Good	Zoo	Euthanasia	Fracture (femur); pneumonia;
38	401–10	Ostrich	J	M	Good	Zoo	Spontaneous	Omphalitis; typhlocolitis;
39	1082–10	Ostrich	N	M	Good	Zoo	Spontaneous	Anasarca, renal mineralization;
40	1086–10	Ostrich	E	M	n. r.	Zoo	Spontaneous	Anasarca;
41	1531–10	Rhea	A	F	Poor	Farm	n. r.	Enteritis (*Lawsonia* sp.);
42	312–11	Ostrich	E	n. r.	Poor	Zoo	Spontaneous	Anasarca;
43	313–11	Ostrich	E	n. r.	Good	Zoo	Spontaneous	Anasarca; yolk sac inflammation;
44	314–11	Ostrich	E	n. r.	Good	Zoo	Spontaneous	Anasarca; yolk sac inflammation;
45	558–11	Ostrich	J	F	Good	Farm	Spontaneous	Subcutaneous hemorrhage (neck, upper leg); hepatic lipidosis;
46	575–11	Ostrich	J	M	Moderate	Zoo	Euthanasia	No significant morphologic lesions contributing to death;
47	901–11	Rhea	A	M	Good	Zoo	Spontaneous	Degenerative cardio-myopathy; thyroid adenoma; degenerative joint disease;
48	1025–11	Rhea	A	F	Poor	Zoo	Euthanasia	Articular gout; renal adenoma; multifocal muscular hemorrhage (legs); endoparasitism;
49	1078–11	Ostrich	A	F	Good	Zoo	Spontaneous	Granulomatous splenitis and thymitis (Mycobacteriosis);
50	1405–11	Ostrich	J	F	Good	Zoo	Spontaneous	Hemorrhages (sternum, legs); bilateral sharp dissection of skin with opening of joints (tarsometatarsus);
51	1429–11	Ostrich	A	F	Good	Zoo	Spontaneous	Endocarditis; chronic hepatitis; septicemia;
52	399–12	Ostrich	A	F	Good	Zoo	Spontaneous	Hemorrhages (hip, hepatic serosa and parenchyma); endoparasitism;
53	1472–12	Emu	A	F	Good	Farm	Euthanasia	Encephalitis; intraosseous fibrosarcoma; pathological fracture; hepatitis; nephritis;
54	124–13	Ostrich	A	M	Good	Zoo	Spontaneous	Encephalitis; subcutaneous and muscular hemorrhages (head, neck);
55	277–13	Emu	A	M	Good	Zoo	Euthanasia	Uveitis; retinal detachment; conjunctivitis; subcutaneous and intra-coelomic hemorrhages (thorax wall); nephritis; spongy degeneration (brain);
56	300–13	Ostrich	A	F	Good	Farm	Spontaneous	Aortic aneurysm; endoparasitism;
57	353–13	Ostrich	A	F	Good	Zoo	Spontaneous	Rhinitis (Aspergillosis); hypertrophic cardiomyopathy; arteriosclerosis;
58	438–13	Ostrich	J	M	Poor	Farm	Spontaneous	Intestinal impaction;
59	601–13	Ostrich	A	F	Good	Zoo	Euthanasia	Hepatic lipidosis; granulomatous pneumonia (Aspergillosis); endoparasitism;
60	822–13	Ostrich	J	F	Good	Zoo	Spontaneous	Skeletal muscle degeneration; cardiomyopathy;
61	1033–13	Ostrich	A	F	Good	Zoo	Spontaneous	Epi- and myocarditis; hepatic lipidosis; rhinitis;
62	188–13	Rhea	A	n. r.	Good	Farm	Spontaneous	Parabronchitis (Influenza A); glomerulonephritis; skeletal muscle degeneration;
63	170–13	Emu	A	n. r.	Moderate	Zoo	Spontaneous	Disseminated granulomatous inflammation (Mycobacteriosis); granulomatous tracheitis (Aspergillosis);
64	1405–13	Ostrich	J	M	Poor	Farm	Spontaneous	Intestinal intussusception
65	1406–13	Ostrich	N	M	Poor	Farm	Spontaneous	Enteritis; nephritis;
66	962–14	Rhea	J	M	Cachectic	Farm	Spontaneous	Proventricular calcifications; cachexia;
67	934–14	Ostrich	J	M	Poor	Zoo	Spontaneous	Enteritis; esophagitis;
68	1394–14	Ostrich	J	F	Poor	Farm	Spontaneous	Proventriculitis; rhinitis; endoparasitism;
69	1399–09	Ostrich	A	F	Good	Zoo	n. r.	Serositis; hepatic and renal lipidosis;
70	3554–85	Ostrich	A	F	Good	Zoo	Spontaneous	Cardiomyopathy; rupture of joint capsule and tendons with hemorrhages (metatarsophalangeal joint);
71	4397–73	Ostrich	A	F	Good	Zoo	n. r.	Perforation of thoracic wall;

n. r. = not recorded; F = female; M = male; A = adult; J = juvenile; N = neonatus; E = embryonated egg; DIC = disseminated intravascular coagulopathy

### Histopathology

Each case was reviewed retrospectively, except cases Nos. 7, 8 and 12, because paraffin blocks and sections were not available. Histologic sections, stained with hematoxylin and eosin, were examined. In selected cases additional sections were stained with hematoxylin and eosin (HE) or special stains including periodic acid-Schiff (PAS) reaction, Gram, Congo red, von Kossa´s, Heidenhain's Azan, Ziehl-Neelsen´s stain, Grocott's methenamine and Warthin-Starry silver impregnation according to standard laboratory protocols [26].

### Immunohistochemistry

For the detection of tick-borne encephalitis virus, Borna disease virus, equine herpesvirus, rabies virus, influenza A virus, *Lawsonia intracellularis* and *Listeria monocytogenes*, pathogen-specific antibodies were applied as previously described [27–31], and the avidin—biotin-peroxidase complex method was used according to the manufacturers´ instructions (VECTASTAIN Elite ABC Kit, Vector Laboratories, Burlingame, California). For the detection of *Brachyspira* sp., a direct immunofluorescence test using a polyclonal rabbit antibody against *Brachyspira hyodysenteriae* was applied (kindly provided by Prof. G. Amtsberg, Institute of Microbiology, University of Veterinary Medicine Hannover, Germany).

For the visualization of accumulated pathologic prion protein (PrP), paraffin sections were autoclaved at 121°C for 20 min in citrate buffer, pH 6.0 after dewaxing and incubation for 30 min in 3% H_2_O_2_ in methanol. The PrP-specific primary monoclonal antibodies (mabs) F99/97.6.1 (VMRD, Inc.; Pullman, WA, USA) [32] or mab 6C2 (Central Veterinary Institute Wageningen, NL) [33] were applied in Tris-buffered saline with 10% goat serum and 0.03% sodium azide, at a concentration of 0.2 μg/ml and 0.7 μg/ml and incubated for 2 hours at room temperature. As negative control, sections were incubated with 0.2 μg/ml of a monoclonal antibody directed against glycoprotein GP5 of the porcine reproductive and respiratory syndrome virus (PRRSV) used as a null-mab. As a conjugate, the avidin-biotin-peroxidase complex method was used as described above. For both methods 3, 3′-diaminobenzidine-tetrahydrochloride (DAB, Sigma-Aldrich, Munich, Germany) was used as chromogen. Sections were counterstained with Mayer`s hematoxylin and mounted.

### Western blot

In order to test the binding of mammalian PrP-specific antibodies to ratite-associated PrP, 10% cattle, ostrich and rhea brain homogenates were prepared in Sucrose-DOC-NP40 (0.42 M Sucrose, 0.5% deoxycholic acid sodium, 0.5% Nonidet P40). Samples were mixed with gel loading buffer containing 6% sodium dodecyl sulphate (SDS) and analyzed in a SDS-PAGE as described previously [34]. Mabs F99/97.6.1 and 6C2 were applied as detection antibodies at a concentration of 0.05 μg/ml and 0.07 μg/ml, respectively, which are well established for the diagnostic of transmissible spongiform encephalopathy in ruminants. Both antibodies bind to conserved regions with little variation in the prion protein sequence between ruminants and ratites.

### Polymerase chain reaction

For the detection of genome fragments of *Brachyspira hyodysenteriae* and *Lawsonia intracellularis* polymerase chain reactions (PCRs) were performed as previously described [35].

## Results

Between 1968 and 2014, a total of 71 ratites including 54 ostriches, 5 emus and 12 rheas were submitted for pathological examination. Spontaneous death was recorded in 19 adults, 17 juveniles and 10 neonates and embryonated eggs, whereas 8 adults, 5 juveniles and one neonate were euthanized. For the remaining 11 cases the mode of death was not recorded. Eighty-three percent of the animals (n = 59) were kept in zoological institutions and 17% (n = 12) on private farms in northwestern Germany. Fifty-two percent (n = 37) of the ratites were adults (older than 1.5 years), 32% (n = 23) were juvenile birds (older than 5 days and under 1.5 years of age), and 15% (n = 11) were neonates (under 5 days of age) or embryonated eggs. The body condition was assessed as good in 57% (n = 41), as moderate in 10% (n = 7), and as poor in 17% (n = 15). In 6% (n = 4) of the ratites a cachectic body condition was noted and in 10% (n = 7) the body condition was not recorded. In many animals more than one morphologic change attributing to a different disease process was found.

### Musculoskeletal system

**Fig 1:**
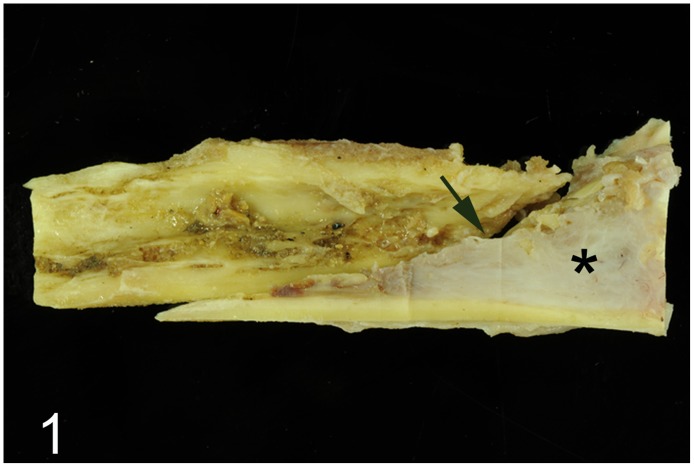
Intraosseous fibrosarcoma, left tarso-metatarsal bone, emu, case No. 53. Adjacent to the gray-white neoplastic tissue that occupies partially the marrow cavity (asterisk) the oblique fracture line of the bone is visible (arrow).

**Fig 2:**
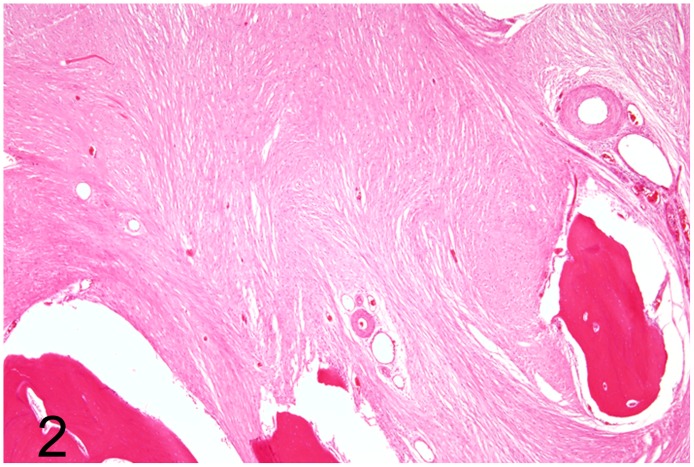
Intraosseous fibrosarcoma, left tarso-metatarsal bone, emu, case No. 53. The fibrosarcoma is composed of spindle-shaped neoplastic cells embedded in abundant amounts of collagen fibers. Hematoxylin and eosin (HE).

Diseases of the musculoskeletal system were diagnosed in 18 (49%) adult animals (case Nos. 3, 5, 6, 8, 13, 21, 23, 32, 47, 48, 52–55, 61, 62, 70, 71). Traumatic injuries to the legs and trunk associated with muscular degeneration, lacerations, hemorrhage and local circulatory disturbances characterized by edema and vascular thrombosis were present in 12 cases (case Nos. 3, 5, 13, 21, 23, 32, 48, 52–54, 70, 71). Of these cases, an emu (No. 53) suffered from an intraosseous fibrosarcoma (Fig 1) associated with a focal oblique pathologic fracture of the left distal tarsometatarsus with concurrent lacerations and hemorrhage in the adjacent muscles and skin. The tumor was composed of interlacing bundles of moderately pleomorphic spindle-shaped cells embedded in a collagen fiber-rich extracellular matrix, showed rare mitotic figures (Fig 2) and caused osteolysis of trabecular bone. Other changes comprised mild focal fibrino-purulent tarsitis and hip arthrosis in one ostrich (case No. 8), articular gout in a rhea (case No. 48), and mild hyaline muscular degeneration (case Nos. 11, 61, 62).

Ten (44%) out of 23 juvenile animals presented with lesions in the musculoskeletal system. Traumatic injuries were observed in six animals (case Nos. 4, 15, 16, 28, 45, 50) including a fractured vertebral column (case Nos. 4, 16) and multiple rib fractures. Swollen costochondral areas of the ribs in case No. 15 were suggestive of rickets. Slipped tendons (perosis) with lateral displacement of the gastrocnemius tendon and deformation of the distal tibiotarsal and tarsometatarsal bones were observed in ostriches Nos. 12 and 14. Two animals exhibited hyaline muscular degeneration (case Nos. 26, 28) of unknown cause. Ostrich No. 60 showed widespread hyaline degenerations of the musculus gastrocnemius, musculus extensor metacarpi radialis, musculus iliotibialis lateralis and musculus elevator caudalis as well as a degenerative cardiomyopathy associated clinically with severe depression after transportation. One neonatal ostrich (case No. 37) displayed a traumatic fracture of the femur.

### Digestive system

**Fig 3:**
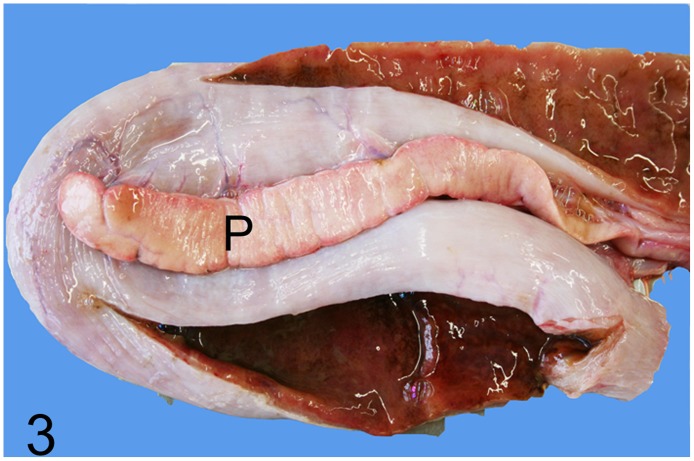
Proliferative enteritis caused by *Lawsonia intracellularis*, intestine, rhea, case No. 41. Red-brown discoloration of the intestinal mucosa with fleshy appearance of the duodenal wall. P = pancreas.

**Fig 4:**
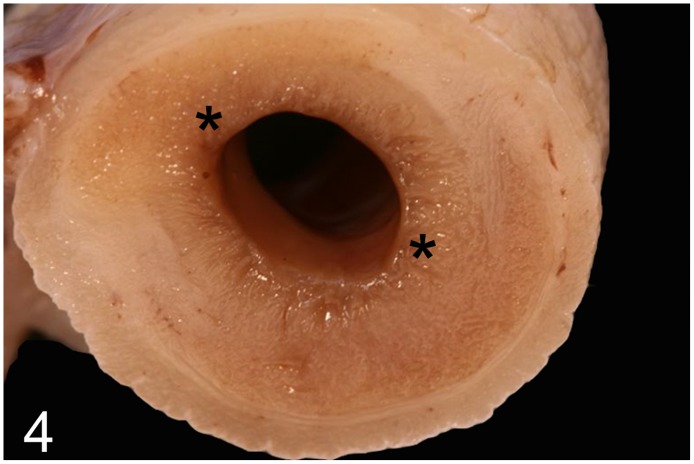
Proliferative enteritis caused by *Lawsonia intracellularis*, intestine, rhea, case No. 41. Marked thickening of the intestinal mucosa (asterisks).

**Fig 5:**
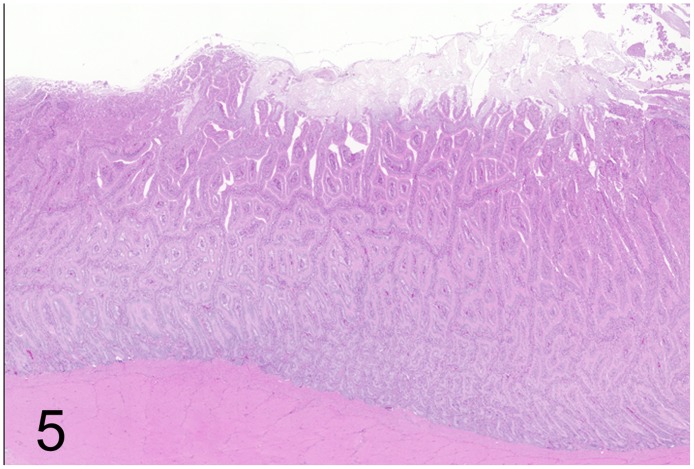
Proliferative enteritis caused by *Lawsonia intracellularis*, intestine, rhea, case No. 41. Subgross magnification reveals severe elongation of the intestinal crypts. HE.

**Fig 6:**
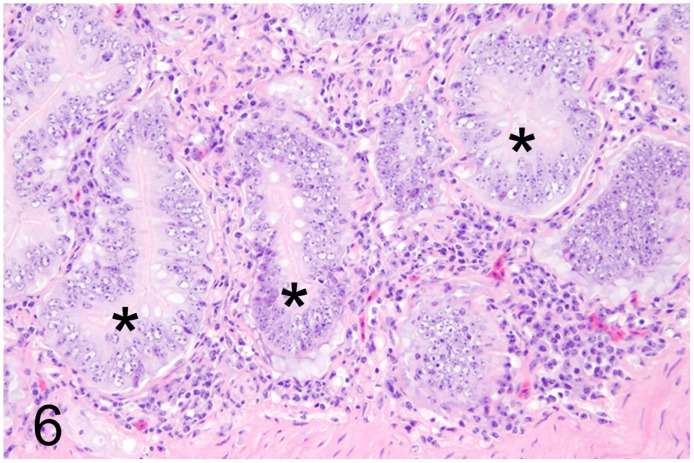
Proliferative enteritis caused by *Lawsonia intracellularis*, intestine, rhea, case No. 41. Severe crypt hyperplasia (asterisks) and moderate inflammatory infiltration of the adjacent lamina propria. HE.

Disorders of the alimentary system were observed in 17 (46%) out of 37 adult animals (case Nos. 1, 3, 6, 17, 18, 21, 22, 32, 41, 48, 51–53, 56, 59, 61, 69). In one adult rhea (case No. 41), proliferative enteritis with marked thickening of the mucosa (Figs 3 and 4), liquid dark brown intestinal content, infiltration of heterophils, macrophages, lymphocytes and plasmacells, as well as severe hyperplasia of intestinal crypts were found (Figs 5 and 6). Although Warthin-Starry silver impregnation and immunohistochemistry for *Lawsonia intracellularis* antigen failed to detect the pathogen, PCR identified genome fragments of *Lawsonia intracellularis* in samples taken from the small intestine. Severe enteritis of unknown cause was diagnosed in ostrich No. 6, and in case Nos. 48, 52, 56 and 59 intraluminal nematodes (not further identified) without inflammatory reactions were observed. Hepatic lesions in adult ratites comprised moderate to severe randomly distributed lympho-histiocytic and lympho-plasmacytic hepatitis (case Nos. 21, 51, 53) as well as mild to severe diffuse hepatic lipidosis (case Nos. 3, 32, 59, 61, 69).

**Fig 7:**
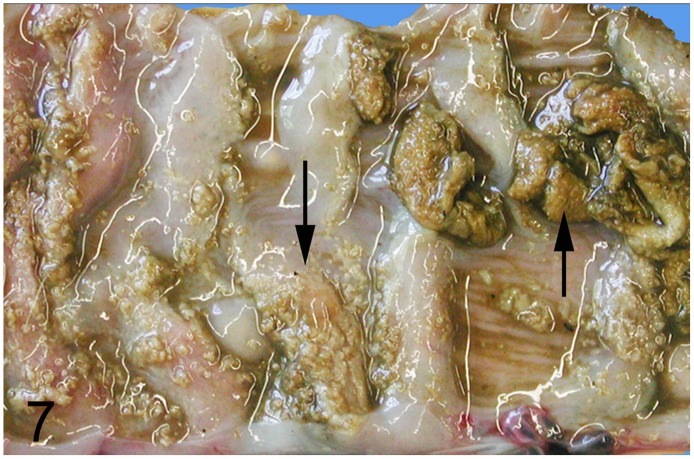
Fibrino-necrotizing typhlocolitis caused by *Brachyspira* sp., intestine, rhea, case No. 31. Brown-greenish tinged fibrin attached to the cecal mucosa (arrows).

**Fig 8:**
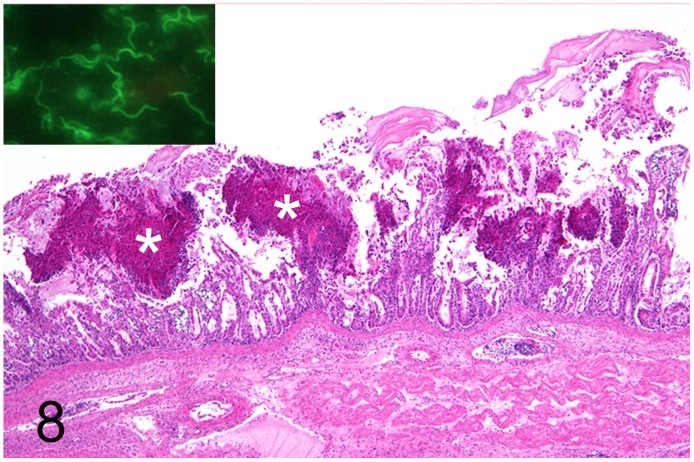
Fibrino-necrotizing typhlocolitis caused by *Brachyspira* sp., intestine, rhea, case No. 31. Multifocal fibrino-necrotic mucosal lesions (asterisks). HE. Inset: Immunolabelling of spirochetes using a polyclonal antiserum against *B*. *hyodysenteriae*. Immunofluorescence.

Disorders of the alimentary system were observed in 10 (43%) out of 23 juvenile animals (case Nos. 9, 11, 31, 38, 58, 64–68). Gastric lesions included non-purulent inflammation of the glandular stomach (case No. 9), purulent proventriculitis associated with numerous intralesional nematodes (not further identified) in the lamina propria mucosae (case No. 68) and multifocal mineralization of the mucosa (case No. 66). Intestinal inflammation consisted of catarrhal and fibrinous enteritis (case No. 65, 67) and catarrhal typhlocolitis (case No. 38). One rhea (case No. 31) suffered from fibrino-necrotizing typhlocolitis (Figs 7 and 8). Warthin-Starry silver impregnation applied on the tissue sections and a *Brachyspira*-immunofluorescence assay applied on feces revealed countless tortuous bacteria of up to 25 μm in length (Fig 8). Genome fragments of *Brachyspira* (*B*.) sp. were detected by PCR. An intestinal intussusception with associated necrotizing and ulcerative inflammatory changes and intralesional bacterial colonies was observed in ostrich No. 64. Ostrich No. 58 showed severe large intestinal impaction of unknown cause with subsequent fibrinous serositis. Perforation of the muscular stomach wall due to ingestion of a sharp foreign body associated with severe purulent and necrotizing inflammation of the gastric wall and serosa was detected in one neonatal rhea (case No. 24).

### Cardiovascular system

**Fig 9:**
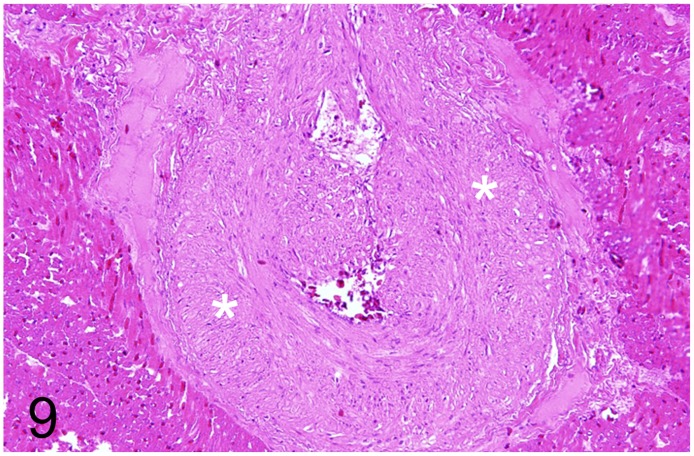
Hypertrophy and hyperplasia of the tunica media, cardiac artery, rhea, case No. 22. Marked increase in thickness of the tunica media (asterisks). HE.

Diseases of the cardiovascular system were diagnosed in 17 adult animals (46%). Myocardial lesions were found in seven and vascular changes in ten animals, respectively. In two ostriches and one rhea (case Nos. 2, 47, 60) a degenerative cardiomyopathy with extensive hyaline degeneration of cardiomyocytes and histiocytic inflammation was observed. In one ostrich (case No. 51), a severe chronic valvular endocarditis with thrombus formation and intralesional Gram-positive bacteria was detected. Ostrich No. 61 showed a focal moderate purulent epi- and myocarditis. A female ostrich (case No. 57), weighing 80 kg, displayed a severe left ventricular hypertrophic cardiomyopathy with an absolute heart weight of 1.3 kg. Streak-like changes in the aortic intima consisting of fibrous tissue were interpreted as arteriosclerotic change. Morphological findings indicating decompensation such as left atrial hypertrophy and dilatation, chronic pulmonary congestion or effusions were not recorded. A 15-year-old female ostrich (case No. 56), weighing 146 kg, died of hypovolemic shock after rupture of a dissecting abdominal aortic aneurysm associated with perivascular hematomas, extensive granulation tissue and thrombosis of periaortic blood vessels. Adjacent to the rupture rims, the aortic wall showed multifocal mural calcifications and necrosis. A moderate focal granulomatous myocarditis was diagnosed in the same animal, but special stains (Ziehl-Neelsen´s stain, PAS-reaction) failed to detect intralesional pathogens including acid-fast bacteria. In eight cases (case Nos. 6–8, 19, 20, 22, 23, 33) a gradually variable hypertrophy and hyperplasia of the tunica media and proliferation of smooth muscle cells in the tunica intima of arterial vessel walls was diagnosed in various organs (Fig 9). In one ostrich (case No. 6), disseminated intravascular coagulopathy associated with multifocal intravascular fibrin thrombi of undetermined cause was seen. In one juvenile ostrich (case No. 9), disseminated intravascular coagulopathy associated with meningoencephalitis and focal cortical encephalomalacia most likely caused by hypoxia was found.

### Nervous system

**Fig 10:**
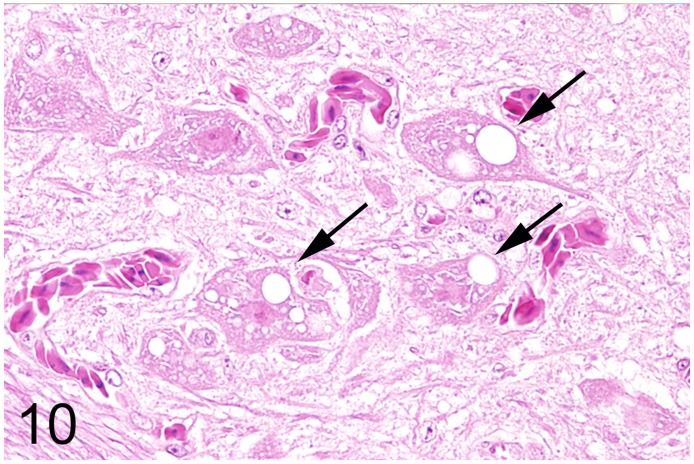
Spongy degeneration, brain, ostrich, case No. 19. Oval to spherical vacuoles of variable diameter (arrows) are present in the perikaryon of several neurons of the red nucleus. HE.

**Fig 11:**
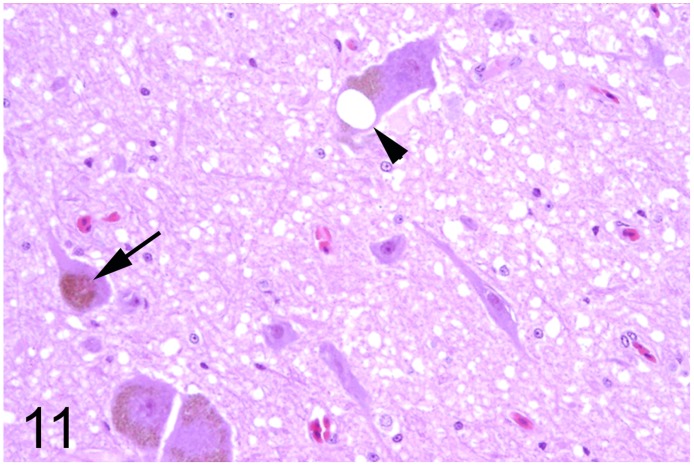
Spongy degeneration and lipofuscin deposition, brain, ostrich, case No. 34. Oval vacuole (arrowhead) and deposition of a coarse granular golden-brown pigment (arrow) in the perikaryon of some brain stem neurons. HE.

**Fig 12:**
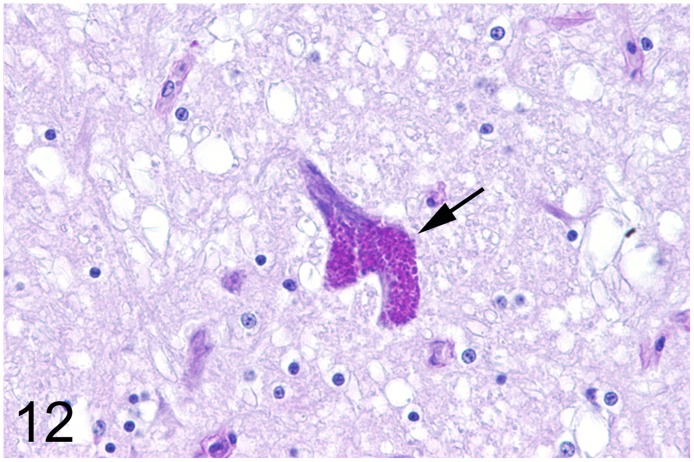
Spongy degeneration and lipofuscin deposition, brain, ostrich, case No. 34. PAS-positive coarse granular pigment deposition suggestive of lipofuscin (arrow) in a brain stem neuron. PAS-reaction.

In 13 (35%) out of 37 adult ratites lesions of the brain were detected. In 6 ostriches (case Nos. 7, 8, 17, 19, 20, 34) and two emus (case Nos. 18 and 55) an encephalopathy with spongy degeneration of various regions of the brain was found. Reported clinical signs in ostriches Nos. 7, 8, 17 and 19 that were necropsied between 1986 and 1993 comprised slowly progressive ataxia with disturbances of balance, depression and anorexia. Ostrich No. 20 showed mild ataxia and possible blindness. In addition, ostriches Nos. 17 and 19 exhibited unusual twisting of the neck and increasing sternal recumbency. In animals Nos. 34 and 55 no nervous signs were noticed. Histological findings in ostriches Nos. 17 and 19 consisted of mild to moderate numbers of round to oval clear vacuoles in the perikaryon of neurons of the substantia grisea centralis, nucleus nervi oculomotorii, and nucleus reticularis pontis oralis and in the neuropil of the brain stem (Fig 10). Spongy changes were distributed bilaterally and symmetrically. The lesions of ostriches Nos. 7 and 8 have been described previously in detail [23, 24]. Briefly, severe vacuolation was observed in the perikaryon of neurons of the nucleus ruber, nucleus vestibularis and nucleus reticularis, and in the neuropil of the brain stem. Congo red stains showed no accumulation of amyloid in the brains associated with spongiosis (case Nos. 18, 20, 33 and 55). In addition, a fine granular, autofluorescent, PAS-positive and acid-fast pigment was observed in the perikaryon of numerous neurons in the same animals interpreted as lipofuscin. In animals Nos. 34 and 55 single vacuoles admixed with a similar pigment were present in the perikaryon of some neurons of the brain stem (Figs 11 and 12). The immunohistochemical evaluation of ostriches Nos. 17, 19, 34 and emu No. 55 failed to demonstrate specific PrP accumulations. Signals observed after incubation of the sections with a null-mab directed against GP5 of PRRSV were indistinguishable from those observed after incubation with both PrP-specific mabs. Analysis of the binding affinity of the two PrP-specific mabs generally used for the transmissible spongy encephalopathy diagnostics in ruminants (F99/97.6.1 and 6C2) did not reveal PrP-specific bands to verify the binding of both antibodies to ratite PrP, although especially in the case of mab 6C2, the binding epitope within the PrP displays no mutation between ruminant and ratite PrP. In contrast, PrP-associated bands were visible for the cattle brain sample loaded as a control (data not shown).

One emu (case No. 53) and one ostrich (case No. 54) with a clinical history of acute trauma suffered from inflammatory lesions in the brain. In emu No. 53 moderate multifocal perivascularly accentuated granulomatous encephalitis was detected predominantly in the telencephalon. However, Ziehl-Neelsen´s stain failed to detect intralesional acid-fast bacteria. In ostrich No. 54 moderate multifocal perivascular lympho-histiocytic and plasmacytic encephalitis predominantly in the cerebellum and in the brain stem was recorded. Immunohistochemical evaluations for tick-borne encephalitis virus, Border disease virus, equine herpesvirus, rabies virus, influenza A virus and *Listeria monocytogenes* antigens remained negative in this case. In ostriches Nos. 49, 52 and 59, mild to moderate lipofuscin depositions were observed in some neurons of the brain stem.

In five (22%) out of 23 juvenile ratites lesions of the brain were detected. Spongy degeneration was found in three juvenile male ostriches (case Nos. 12, 29, 30). According to a previous report [24], ostrich No. 12 that died in 1989 showed clinically slowly progressive ataxia, including disturbances of balance and loss of appetite. Lesions were characterized by severe vacuolation of the neuropil and few neurons of the brain stem and medulla oblongata [24]. Ostriches No. 29 and 30, both in a cachectic nutritional status, presented with clinical signs of apathy, somnolence and opisthotonus. Mild intraneuronal vacuolizations in Purkinje cells, the cortex cerebri and neuropil of the brain stem were found. Immunohistochemical evaluation failed to demonstrate specific PrP accumulations. Congo red stain did not show accumulation of amyloid in brain sections with spongiosis (case Nos. 29 and 30).

In ostrich No. 9 multifocal meningoencephalitis and focal cortical encephalomalacia was found in association with disseminated intravascular coagulopathy. One rhea (case No. 27) displayed severe diffuse granulomatous inflammation of the choroid plexus without demonstrable pathogens including acid-fast bacteria using PAS-reaction and Ziehl-Neelsen´s stain.

### Respiratory system

**Fig 13:**
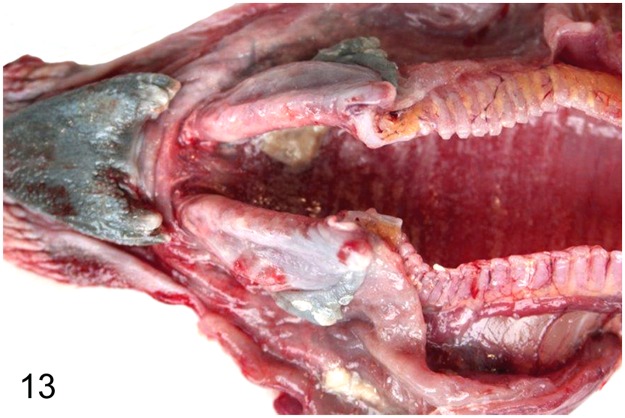
Avian influenza, larynx and trachea, rhea, case No. 62. Severe necro-suppurative laryngo-tracheitis with diffuse hyperemia and numerous white, necrotic foci.

**Fig 14:**
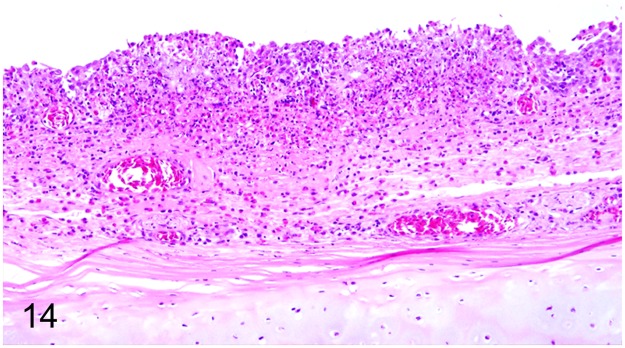
Avian influenza, trachea, rhea, case No. 62. Severe, diffuse, necro-suppurative tracheitis. HE.

**Fig 15:**
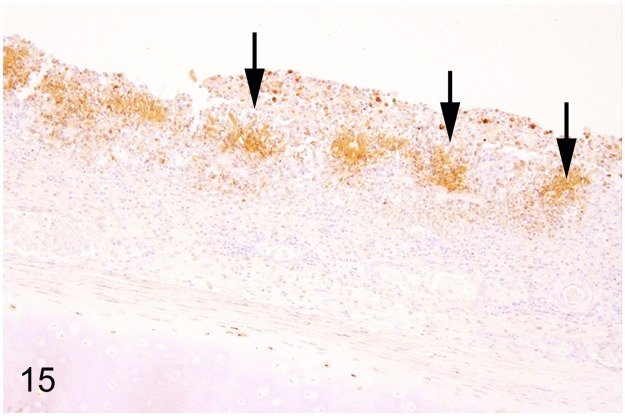
Avian influenza, trachea, rhea, case No. 62. Intralesional immunolabelling of influenza A virus nucleoprotein (arrows). Immunohistochemistry.

Seven (19%) out of 37 adult ratites showed significant changes of the respiratory system. Findings included mycotic rhinitis with numerous vascular thrombi in an ostrich (case No. 57) due to *Aspergillus* sp. and purulent to necrotizing rhinitis (ostrich No. 61). A rhea (case No. 62) with a history of respiratory distress showed purulent laryngitis, multifocal to coalescing suppurative to necrotizing laryngo-tracheitis (Figs 13 and 14) and lympho-plasmacytic parabronchitis. Immunohistochemically intralesional influenza A virus nucleoprotein (Fig 15) was detected in trachea and lung. Molecular analysis performed at the Veterinary State Reference Laboratory revealed subtype H7N7 of influenza A virus. Furthermore, three ostriches (case Nos. 1, 34, 59) suffering from severe granulomatous pneumonia with intralesional septated fungal hyphae indicative of *Aspergillus* sp. were also recorded. One ostrich (case No. 17) showed a purulent aerosacculitis.

In the juvenile ostrich No. 68 nematodes (not further specified) were detected in the nasal cavity associated with a severe diffuse ulcerative and purulent inflammation caused by a secondary bacterial infection. Another ostrich (case No. 30) had a severe granulomatous and necrotizing rhinitis with fungal hyphae, most likely *Aspergillus* sp. In one neonatal ostrich (case Nos. 37) interstitial lympho-histiocytic pneumonia of unknown etiology was observed.

### Urogenital system

Morphological changes of the urogenital system were present in seven (19%) out of 37 adult ratites and included mild multifocal lympho-histiocytic interstitial nephritis (case Nos. 21, 53, 55), multifocal tubulonephrosis and tubulonecrosis (case No. 2), proliferative glomerulonephritis (case No. 62) and a renal adenoma in an adult rhea (case No. 48). Cystic lesions in the testicle without other associated lesions were detected in an ostrich (case No. 3). Mild multifocal tubulonephrosis was diagnosed in one juvenile ostrich (case No. 29.).

### Systemic diseases

**Fig 16:**
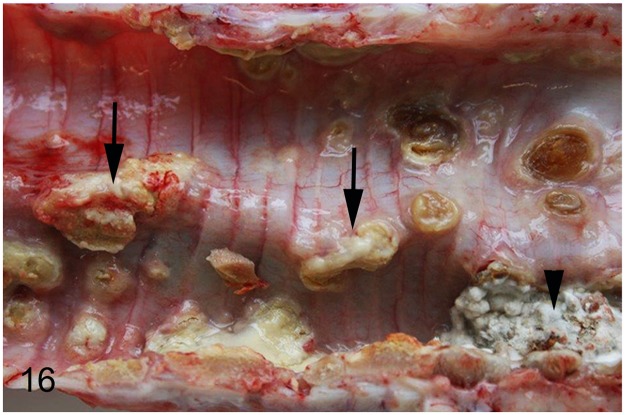
Avian mycobacteriosis and aspergillosis, trachea, emu, case No. 63. Multifocal yellow granulomas (arrows) in the trachea caused by *M*. *avium* subsp. *avium* and focal white granuloma caused by *Aspergillus* sp. (arrowhead).

**Fig 17:**
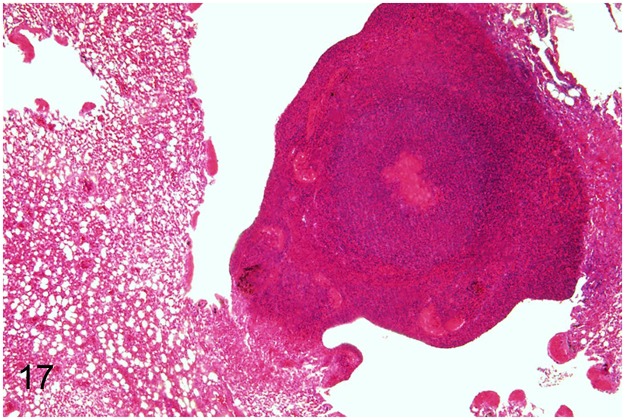
Avian mycobacteriosis and aspergillosis, lung, emu, case No. 63. Necro-granulomatous pneumonia. HE.

In three (8%) adult animals (case Nos. 34, 49, 63) systemic diseases characterized by granulomatous inflammations were observed. Using Ziehl-Neelsen´s stain intralesional acid-fast bacterial rods were found in all cases. In ostrich No. 34, granulomas were present in conjunctiva, lung, spleen, liver, ovaries and serosal surface of the body cavity. In ostrich No. 49 granulomas were observed in spleen and thymus. Microbiologic examination failed to isolate *Mycobacterium* (*M*.) sp. in both cases. In emu No. 63, housed together with wallabies, *M*. *avium* subsp. *avium* was identified in granulomatous lesions of trachea, lung, spleen and liver (Figs 16 and 17). One of the wallabies was also reported to be infected with the same bacterium. In two animals (case Nos. 34, 63), an additional granulomatous mycotic infection in lung, air sacs and serosal surface of the body cavity with intralesional septated hyphae most probably *Aspergillus* sp. was observed. The juvenile ostrich No. 30 presented granulomatous inflammation with similar intralesional fungi in nasal cavity, esophagus and body cavity.

**Fig 18:**
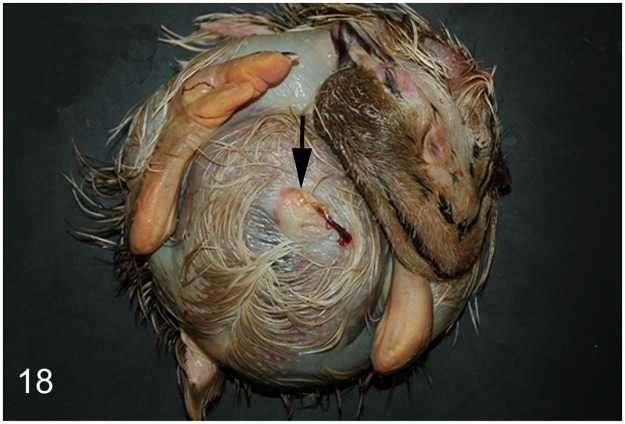
Neonatal death, subcutis, ostrich, case No. 35. Generalized subcutaneous edema (anasarca) and purulent omphalitis (arrow).

Seven (64%; case Nos. 35, 36, 39, 40, 42–44) out of 11 embryonated eggs or neonatal ostriches displayed anasarca, a generalized subcutaneous edema. In three animals, there was also a moderate to severe edema of the musculature present. In two cases (Nos. 35, 36), purulent omphalitis (Fig 18) and in two additional ostriches (case No. 43, 44) necro-suppurative inflammations of the yolk sac were observed, both lesions most likely caused by bacterial infections. In ostriches Nos. 39 and 40, mild mineralized concrements in renal tubuli were observed, and in ostriches Nos. 43 and 44, mild mineralizations were detected in the proventricular mucosa.

### Miscellaneous disorders

A thyroidal adenoma was found in an adult rhea (case No. 47). Granulomatous dermatitis occurred in an adult ostrich (case No. 57), however, Ziehl-Neelsen´s stain and PAS-reaction failed to detect intralesional acid-fast bacteria and other pathogens. In two ratites (case Nos. 34, 55), a granulomatous and proliferative conjunctivitis with intralesional acid-fast bacterial rods and a lympho-plasmacytic conjunctivitis were documented. In one emu (case No. 18), a fibrino-purulent inflammation of the body cavity was found and *Haemophilus* sp. was isolated from the lesion. Emu No. 55 showed a bilateral retinal detachment associated with lympho-plasmacytic uveitis of unknown etiology.

## Discussion

This retrospective survey was conducted in order to elucidate the principal causes of disease in various ratite species, particularly ostriches, held in captivity. The majority of the animals (83%) were kept in zoological institutions. Although half of the farmed ratites of this study were emus and rheas, in Germany mainly ostriches are used for farming purposes. The predominantly affected organ systems in both, adults and juveniles, were the musculo-skeletal, digestive and central nervous systems. In adults, the cardio-vascular system ranked also among the most frequently altered organs. In most eggs and neonates, morphological findings were suggestive of circulatory failure. The predominant and most salient lesions will be discussed below.

Lesions of the legs, neck and trunk with associated hemorrhage, muscular laceration and degeneration, most likely caused by traumatic impacts, were the most prominent cause of disease in adult and juvenile ratites. The high number of traumatic injuries, supported by clinical evidence of trauma, may be associated with housing situations on farms and in zoological institutions. Femoral and tibiotarsal fractures frequently result in severe hemorrhages followed by hypovolemic shock and death. The tibiotarsus is probably the most trauma-exposed bone of the ratite skeleton [36]. In addition, tarsometatarsal fractures are usually open with the risk of infection, and prolonged recumbency often results in muscular degeneration [12]. With the exception of emu No. 53, no evidence of predisposing conditions for pathological fractures was observed. However, other mechanisms or underlying disorders, e. g. metabolic changes, cannot be ruled out completely and would require additional investigations including determination of calcium and phosphor values in the blood and bones. In emu No. 53, a spontaneous pathological fracture due to an intraosseous fibrosarcoma was found. Skeletal fibrosarcomas arise from connective tissue stroma in either the medullary cavity, as in this case, or the periosteum. Fibrosarcomas of different locations are among the most frequently observed in pet birds [37]. However, this variant has not been described in ratites before.

In ostrich No. 60, hyaline muscular degeneration (Zenker´s degeneration) of various skeletal muscles was observed. With respect to the clinical history of depression after transportation, this finding is suggestive of capture myopathy as possible cause of disease and death. Transportation is considered as one of the most stressful procedures in managing ratites [38, 39]. Because their center of gravidity is rather high above the ground [38], these animals have difficulties in achieving balance, particularly during shipping on trucks. In addition, ratites are very nervous birds making their transportation on trucks more complex and stressful than for food and companion animals like cattle and horses [38, 39]. However, in ostrich No. 60, an additional degenerative cardiomyopathy was present. As exertional myopathy caused by transportation is reported to be restricted to skeletal muscles [10] differential diagnoses for degenerative skeletal and cardiac muscular disorders have to be considered, e. g. ionophore intoxication [40]. In ostriches, myocardial damage has not been reported with selenium deficiency [41].

In two juvenile ostriches, slipped tendon characterized by lateral displacement of the gastrocnemius tendon was diagnosed which occurs in all ratite species, but ostriches and emus are preferentially affected [14]. Causes include genetic predisposition and malnutrition (manganese, biotin, pantothenic acid, folic acid and choline deficiency) as possible predisposing factors [14, 42]. Elevated selenium levels as well as a lack of copper and calcium are suggested to play a pathogenetic role in ostrich chicks [43]. In the presented cases, no essential nutrients or mineral values were determined.

Gastro-intestinal disorders were the second most common cause of diseases in adult and juvenile ratites of this study. Common factors causing enteritis in chicks and juveniles are failure to establish a balanced gut flora, the use of antibiotics, lack of fibers in the diet, hypothermia, excessive coprophagy and poor hygiene [11, 44]. Bacterial pathogens associated with enteritis include *E*. *coli*, *Salmonella* sp. *Klebsiella* sp., *Aeromonas* sp., *Pseudomonas* sp., certain strains of *Campylobacter jejuni*, and *Clostridium* (*C*.) *perfringens* may also be involved [11, 13, 14, 44–46]. In the current study, *Lawsonia intracellularis* (case No. 41), the causative agent of porcine proliferative enteropathy [47], was identified as one specific enteric pathogen that was identified as one specific enteric pathogen that may also affect emus among other species [18]. The intestinal changes in rhea No. 41 correspond to the described lesions and PCR confirmed the presence of *Lawsonia intracellularis*, which represents the first case in this ratite species. In the 3-month-old rhea No. 31, *Brachyspira* sp. was identified as the cause of a severe fibrino-necrotizing typhlocolitis. The spirochete *Brachyspira* is capable of causing enteric disease in avian, porcine and human hosts, amongst others [48] and rheas naturally colonized with *B*. *hyodysenteriae* commonly show severe typhlitis and mortality can reach 80% [49, 50]. Stress after shipping is regarded as a contributing factor [49].

Intussusception is often seen in ostrich chicks and juveniles following gastric impaction [11]. Other causes of intestinal intussusception are chronic diarrhea, frequently associated with other intestinal disorders such as gastro-intestinal displacement, torsion and volvulus, and abnormal peristaltic contractions due to unknown reasons [14]. In the described ostrich (case No. 64), no underlying cause was found.

Ostrich No. 68 suffered from gastric nematodiasis and associated inflammation of the glandular stomach, most likely leading to a poor body condition. Nematodes such as *Libyostrongylus* (*L*.) *douglassii*, *L*. *dentatus* and *L*. *magnus* are found in glands of the proventriculus and under the coilin layer of the gizzard of ostriches [45, 51] and rheas [11]. Only the former two have been reported outside Africa [52] with *L*. *douglassi* also infecting ostriches in Germany [22] and The Netherlands [53]. The inflammation inhibits gastric secretions and hinders digestion. The disease is called “vrootmag” or “rotten stomach” as food decays within the stomach and can lead to weight loss and high mortality in ostrich chicks and juveniles potentially associated with economic losses in ratite farming [12, 14, 51].

In one young rhea (case No. 24), perforation of the muscular stomach was caused presumably by a foreign body. Ingestion of foreign bodies is a common problem in ratites [14, 44] because these animals will swallow anything that fits into their mouths, and their curiosity and enquiring eye ensure that they will find many unusual items in their enclosure [14].

The adult ostrich No. 56 died from a dissecting aortic aneurysm with degenerative changes of the aortic wall which probably represented a predisposing factor along with assumed hypertension. According to the literature, no atherosclerotic changes of the aorta are associated with the rupture site. However, copper deficiency that predisposes to defective elastin is suggested as a possible cause [54–57] which is also described in turkeys [58]. Other causes might be systemic hypertension and a genetic predisposition [55]. The other vascular lesions identified in this study (case Nos. 6–8, 19, 20, 22, 23 and 33) consisted of gradually variable thickening of the tunica media of arteriolar vessel walls of parenchymatous organs like kidney and spleen. These changes did not resemble atherosclerosis [59, 60], and were regarded most likely as incidental findings.

In ostrich No. 57, a considerable hypertrophy of the myocardium was found with a relative heart weight of 1.63%. The reference range of the absolute heart weight in ostriches of about 122 kg body weight is reported to be 1.054 kg +/- 172 g corresponding to a relative heart weight of 0.86% (+/- 0.14%). The cause of the cardiac hypertrophy in the study case remains undetermined, however, evidence of decompensation was not found. The fibrous streaks in the aortic intima may be pathogenetically related to the cardiac disorder or represent a separate disease process of unknown origin. Abnormal jets of blood flow or turbulences have to be considered as possible causes.

The principal change encountered in the nervous system was a spongy encephalopathy in six adult ostriches (case Nos. 7, 8, 17, 19, 20, 34), two emus (case Nos. 18, 55) and three juvenile ostriches (case nos. 12, 29, 30). The clinical and morphological findings of three of these animals (case Nos. 7, 8, 12) from two different zoological collections in Germany have already been described [23, 24]. In case Nos. 17 and 19, the lesions were observed bilaterally in the brain stem and are regarded most likely as cause of the clinical nervous signs. Spongy degeneration in domestic animals may affect either gray or white matter, or both, in a bilateral and symmetrical fashion and is caused by cytotoxic edema resulting in intracellular fluid accumulation due to disrupted osmoregulation [61]. Possible underlying mechanisms include toxins, gene defects and infectious as well as metabolic processes. The cause of the spongy degeneration observed in the present study remains undetermined. The temporal coincidence of case Nos. 7, 8, 12, 17 and 19 during the years 1986 to 1993 with the emergence of bovine spongiform encephalopathy in the United Kingdom and the subsequent occurrence throughout many European countries since 1990 raised the possibility of a causal link between both diseases. So far all investigations failed to proof a link between transmissible spongiform encephalopathy and the brain lesions in ratites [62]. Although the presented cases share clinical and morphological features described for transmissible spongiform encephalopathies in mammalian species, the application of two different PrP-specific antibodies that are routinely used for the diagnostics of ruminant transmissible spongiform encephalopathy revealed no accumulation of pathological PrP. However, the binding affinity of the applied antibodies to ratite PrP could not be irrevocably demonstrated. In contrast to domestic animals [63], there is little information concerning spongy degeneration in the central nervous system of birds. Indications for inherited, metabolic, toxin- or dietary-related disturbances were not found in the present cases. In ostriches Nos. 34 and 55, marked lipofuscin deposits in neurons and the absence of nervous signs suggest an age-related spongy degeneration. Neuronal vacuolation in case No. 29 may possibly be related to the poor nutritional condition that might be caused, at least in theory, by nutritional deficiencies, metabolic disorders and intoxications [62].

In emu No. 53 and ostrich No. 54, inflammatory lesions of the brain were found that were associated with multiple subcutaneous and muscular hemorrhages in the ostrich. The etiology of the encephalitis remained undetermined in both cases but an infectious agent, most likely a virus, has to be considered.

Respiratory diseases in adult ostriches and rheas included rhinitis (case Nos. 57, 61) influenza A virus infection (case No. 62) and mycotic granulomatous pneumonia (case Nos. 1, 34, 59). Fungal infections of the respiratory organs occur most frequently in neonates and juveniles housed in enclosed facilities and exposed to dust or hay which is alternatively wet or dry [45]. *Aspergillus fumigatus* and other *Aspergillus* sp. are commonly isolated and can also affect older birds, often in conjunction with immunosuppression or concurrent disease [11, 45]. Fungal granulomatous rhinitis restricted to the nose as in case No. 57 has been described rarely in ratites [64]. An adult rhea was infected with influenza A virus H7N7 sharing the reported lesion profile associated with avian influenza infection in other ratite species [13, 65]. Ostriches, emus and rheas are highly susceptible to avian influenza, and various subtypes have been identified including H7N1, H5N9, H5N2, H9N2 and H10N7 [11]. Natural infection with the subtype H7N7 isolated in this study has not been documented in ratites before. The suppurative to necrotizing inflammation of the airways and liver in the presented case may have been aggravated by a secondary bacterial infection.

Similar to described cases [66, 67], a conjunctival manifestation as part of a systemic avian tuberculosis was found in ostrich No. 34. In case of an isolated mycobacterial lesion of the conjunctiva an entry of the organism through an eyelid defect has to be considered.

Three adult ratites showed granulomatous lesions in multiple organs with intralesional acid-fast positive bacteria indicative of mycobacteria, presumably *M*. *avium* subsp. *avium*. The susceptibility of ratites to avian tuberculosis remains unknown. Nonetheless, the disease has been described in emus and rheas and occasionally in ostriches [68]. It occurs more commonly in multispecies exhibition places like zoos than on single species farms [10]. Tuberculosis has potentially great economic significance, first due to loss of valuable birds and second due to the risk of transmission to other avian and mammalian species [66]. The distribution of granulomatous lesions (trachea, spleen and liver) in emu No. 63 suggests an oronasal infection as the possible portal of entry. This animal was housed in a zoo together with wallabies, one of which was infected with *M*. *avium* subsp. *avium*.

In embryonated eggs and neonates, the most common findings were generalized subcutaneous edema and inflammation of the yolk sac or umbilicus. These lesions are indicative of an improper incubation of the eggs at too high relative humidity. Besides yolk sac retention, bacterial infections of yolk sac or umbilicus are frequently observed in these “wet chicks” [69].

## Conclusion

The high frequency of traumatic injuries in adult and juvenile individuals underlines the susceptibility of ratites to mechanical injuries under housing conditions unlike their natural habitat. The cause and pathogenesis of unusual spongy changes of the brain associated with central nervous signs remain obscure. Particularly, in juvenile animals, bacterial infections of the alimentary tract were an important cause of disease that may be related to husbandry, feeding and other management procedures. Anasarca and omphalitis, the most common findings observed in embryonated eggs and hatchlings, are a worldwide problem occurring in captive ratites most likely caused by improper incubation conditions. The disorders observed in various age groups of captive ratites living in northwestern Germany may help to guide welfare discussions regarding the keeping of these species in farms or zoological institutions.
